# High Value-Added Reutilization of Waste-Printed Circuit Boards Non-Metallic Components in Sustainable Polymer Composites

**DOI:** 10.3390/molecules28176199

**Published:** 2023-08-23

**Authors:** Dechao Hu, Xianghong Zeng, Yinlei Lin, Yongjun Chen, Wanjuan Chen, Zhixin Jia, Jing Lin

**Affiliations:** 1 School of Materials Science and Hydrogen Energy, Foshan University, Foshan 528000, China; msdchu@163.com (D.H.);; 2 Key Lab of Guangdong High Property and Functional Macromolecular Materials, School of Materials Science and Engineering, South China University of Technology, Guangzhou 510640, China; 3 Research Center of Flexible Sensing Materials and Devices, School of Applied Physics and Materials, Wuyi University, Jiangmen 529020, China

**Keywords:** waste-printed circuit boards, reutilization, sustainable polymer composites, electronic wastes

## Abstract

The reutilization non-metallic components from a waste-printed circuit board (WPCB) has become one of the most significant bottlenecks in the comprehensive reuse of electronic wastes due to its low value and complex compositions, and it has received great attention from scientific and industrial researchers. To effectively address the environmental pollution caused by inappropriate recycling methods, such as incineration and landfill, extensive efforts have been dedicated to achieving the high value-added reutilization of WPCB non-metals in sustainable polymer composites. In this review, recent progress in developing sustainable polymer composites based on WPCB non-metallic components was systematically summarized. It has been demonstrated that the WPCB non-metals can serve as a promising reinforcing and functional fillers to significantly ameliorate some of the physical and chemical properties of polymer composites, such as excellent mechanical properties, enhanced thermal stability, and flame retardancy. The recovery strategies and composition of WPCB non-metals were also briefly discussed. Finally, the future potentials and remaining challenges regarding the reutilization of WPCB non-metallic components are outlined. This work provides readers with a comprehensive understanding of the preparation, structure, and properties of the polymer composites based on WPCB non-metals, providing significant insights regarding the high value-added reutilization of WPCB non-metals of electronic wastes.

## 1. Introduction

Recent decades have witnessed rapid development and phenomenal progress in electronic industries, which significantly increased the supply of electrical and electronic products to the public, occurring in tandem with the technological innovation in and continuous falling price of new products [1,2,3]. Moreover, the average service lives of electronic devices have greatly shortened, eventually lead to a staggering increase in electrical and electronic equipment (WEEE) and electronic waste (e-waste) [4,5,6]. It is estimated that 53.6 million tons of e-waste is generated globally per year, and this figure is expected to double by 2050 [7,8]. The environmental pollution caused by e-waste and its resource utilization have become an emerging social problem and created a major challenge to global sustainable development [9,10]. Thus, recycling and eco-friendly reutilization of e-waste has attracted extensive interest from scientific and industrial researchers.

Printed circuit boards (PCB) are integral and indispensable components used in almost all electronic devices, and they have been widely applied in various fields of the electronics industry [11,12,13]. Undoubtably, with the acceleration of electronic product renewing, the generated waste-printed circuit boards (WPCBs), including defective products used in manufacturing process and scrapped products, have also dramatically stacked up [14,15,16,17]. The management rational treatment of WPCB has, thus, become an imminent issue [18]. WPCB includes an approximately 30% metallic fraction (e.g., copper, iron, nickel, antimony, lead, and gold) and a 70% non-metallic component (e.g., thermosetting resins, glass fibers, etc.) [19,20,21]. At present, the separation and recovery of valuable metals have been widely investigated owing to their high added value and high purity. However, most of the non-metallic components used in WPCBs are usually incinerated or landfilled without any effective disposal, ultimately leading to severe resource wastage and great damage to the environment [22,23]. Therefore, exploring feasible and appropriate strategies to achieve eco-friendly reuse of non-metallic components used in WPCBs has far-reaching implications in terms of saving resources and mitigating the risk of environmental pollution. Until now, the researchers have searched for many feasible techniques for recycling WPCB non-metallic materials, such as chemical recycling (pyrolysis, depolymerization) and mechanical recycling. Among these techniques, the mechanical treatment of PCBs is considered to be a more straightforward and effective process, and as-obtained WPCB non-metallic powder could be further applied in construction products, modified asphalt, and polymer composites [24,25,26,27]. In particular, over the past few decades, scientists and engineers have dedicated great efforts to developing ecofriendly polymer composites by incorporating WPCB non-metals into various polymer matrixes, including thermosetting resin (e.g., epoxy resins, unsaturated polyester resin, phenolic resin, etc.), thermoplastic (e.g., polypropylene, poly(vinyl chloride), polyethylene, polyvinyl alcohol, polystyrene, acrylonitrile–butadiene–styrene, etc.), and rubber composites due to their wide availability, low cost, and outstanding environmental protection. It was found that the WPCB non-metallic powder could endow polymer composites with excellent mechanical properties, enhanced thermal stability, and flame retardancy. These pioneering and impressive studies have created new opportunities for the high value-added reutilization of WPCB non-metallic components. Until now, although several significant reviews have summarized the recycling and recovery of non-metallic resources from WPCBs [28,29,30], most researchers focused on broader discussion rather than specifically exploring their high value-added reutilization in polymer composites.

Thus, the primary focus of this work was placed on the high value-added reutilization of WPCB non-metals in various polymer composites, which aims to give a systematic and profound understanding of the composition and structure of WPCB non-metals, the preparation strategies used to create sustainable polymer composites and the structure–properties relationships between WPCB non-metals and polymer composites, as well as their promising potential applications. Finally, the potential uses and remaining challenges of WPCB non-metallic components used in the rational design of high-performance sustainable polymer composites are considered. By systematic discussion of relevant achievements, we hope that this work will provide meaningful and valuable insights into the recycling and high value-added reutilization of non-metallic components of WPCBs.

## 2. Recovery and Characterization of WPCB Non-Metallic Components

Although waste-printed circuit boards only account for about 3% of all produced e-waste, the complex toxic components used in WPCBs, which involve heavy metals and brominated flame retardants, make them hazardous waste that must be treated very cautiously [31,32,33]. Moreover, considering the fact that various valuable metals, polymers, glass fiber, and toxic components were simultaneously integrated in such small volumes, the recovery and recycling of WPCBs become particularly intractable. In general, the compositions of a WPCB are divided into non-metallic components and metallic components [34]. Currently, driven by the economic interests, the recovery of metallic components from WPCBs has attracted extensive attention by using various extraction processes, including leaching, mechanical and hydrometallurgical processing techniques [35,36,37]. For comprehensive reviews of metal recovery from WPCB, readers can refer to the reviews written by Hao et al. [38], Qiu et al. [39], Lu et al. [40], and Akcil et al. [41]. While this review will focus on the recovery techniques used for non-metallic components, which account for about 70 wt% of waste PCBs and still face serious environmental and economic challenges [24]. On the other hand, to better reuse these non-metallic components of WPCB in polymer composites, the detailed characterization of WPCB non-metals is also essential, as they have a decisive influence on the properties of as-obtained sustainable polymer composites. Thus, the recovery techniques and characterization of non-metallic components from WPCBs are discussed below.

### 2.1. Recovery of WPCB Non-Metallic Components

**Figure 1:**
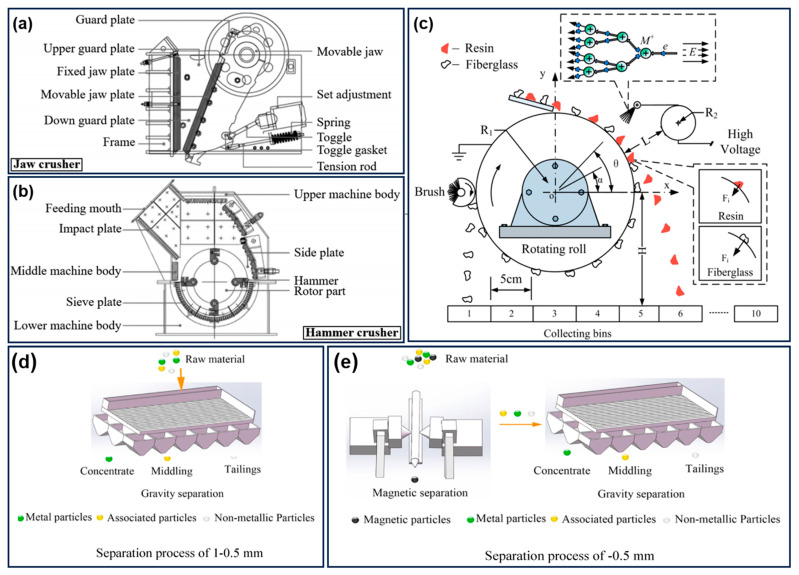
Schematic presentation of conventional WPCB crushers: (**a**) Jaw crusher and (**b**) hammer crusher. Reprinted with permission from [62]. Copyright: 2020, American Chemical Society. (**c**) Diagram of the roll-type corona electrostatic separator. Reprinted with permission from [68]. Copyright: 2014, American Chemical Society. Schematic of recovery process for metal components via (**d**) single gravity separation and (**e**) combined magnetic separation and gravity separation. Reprinted with permission from [61]. Copyright: 2020, Elsevier.

Generally, the recovery strategies used for WPCB non-metallic components include chemical recycling strategies and physical recycling strategies. For chemical recycling, the WPCB are usually depolymerized into some useful molecules via pyrolysis, gasification, supercritical fluids, glycolysis, aminolysis, and alcoholysis [42]. Among these methods, pyrolysis is one of the techniques most commonly used to degrade the resins in WPCB into oils, gases, tar, and glass fibers, which is usually conducted without oxygen or using some inert gas [43,44,45]. The obtained pyrolysis gases exhibit high calorific values, and the pyrolysis oil can be further utilized as the chemical raw material or asphalt modifier. However, the pyrolysis process of WPCB recovery is challenging due to the presence of dibenzofurans, 4-methyl-Benzofluroethane, hydrogen bromide and brominated compounds in pyrolysis oils [46]. Gasification is performed in oxygen, air, or steam at a high temperature, and the gaseous product is similar to that of the pyrolysis process [47,48,49]. Recently, supercritical fluids were widely exploited as a highly efficient means of achieving the metal–non-metal separation in WPCBs, which can destroy the epoxy resin derived from WPCB and produce organic molecules [50,51,52,53]. Typically, the polymer materials can be effectively oxidized within a short time under the action of supercritical fluids, including water, methanol, and ethanol [54,55,56]. Moreover, organic solvents have been employed to dissolve the bromine epoxy resin to efficiently separate the glass fibers and polymer materials in WPCB non-metals [57,58,59]. Unlike the above chemical recycling methods, the physical recycling process is dependent on the differences in terms of physical characteristics between the metallic and non-metallic components of the WPCB. And the shape, size, and size distribution of liberated WPCB have a critical influence on their separation effectiveness. Generally, as shown in Figure 1, the most commonly used separation technologies of WPCB include shape separation, density-based separation, magnetic separation, electrical separation, and electrostatic separation [60,61,62]. For example, density-based separation is usually carried out to separate lighter components from other heavier products based on density difference. However, due to the simultaneous influence of particle shape, its separation efficiency is relatively poor. Electrostatic separation is another promising method used to separate the metals and non-metallic materials, and it has received widespread attention due to its low energy consumption, facile operation conditions, and environmentally friendly characteristics [63,64,65]. Eddy current-based electrostatic separator is generally used to separate thermosetting plastics and non-ferrous metals from the complex mixture via the eddy current and external magnetic field [66,67]. Low-intensity drum magnetic separators can also recover ferrous materials from the non-magnetic fraction. There is no doubt that the physical recovery method is relatively simple, effective, practical, and energy saving, which lays the foundation for the diversified applications of recycled non-metallic products derived from WPCBs. However, there are still some issues related to the physical process of recycling WPCBs. Generally, it is very difficult to completely remove all metals through the physical recycling process, and some residual metals in the as-reclaimed non-metallic components may lead to serious deterioration in the aging properties of polymer composites. Moreover, other than the residual metals, the complex compositions of non-metallic components, including various resin powders, glass fiber, metals derivatives, and even some toxic additives, make their high value-added reutilization even more challenging.

### 2.2. Compositions and Structure of WPCB Non-Metals

The compositions of non-metallic components in WPCB vary depending on the different types of pristine PCBs. In general, non-metallic components in WPCBs are derived from the substrates, accounting for appropriately 70% of PCBs. The choice of substrate materials is closely related to the application of PCBs. For example, epoxy resins are usually utilized in multi-layered PCBs, while the phenolic resins are used in single-layered PCBs [17,69]. As for their application to radio-frequency fields, we usually choose the low-loss Teflon substrates. To endow the PCB substrate with excellent properties, glass fibers or cellulose fibers are usually incorporated to reinforce the resins. Moreover, some inorganic materials are present, including mineral filler, alumina, and other oxides. Among these materials, the glass-fiber reinforced epoxy resins are the most used substrates. In our previous work, to realize the better reutilization of WPCB non-metallic components, the compositions and characteristics were comprehensively investigated [23]. It was found that the thermosetting resin was mainly tetrabromobisphenol A epoxy resin, and the glass fibers were largely separated from resin particles after ball milling, which shows great potential as the strength enhancer of sustainable polymer composites. Furthermore, the presence of residual metal, such as Cu and Fe, may lead to serious aging of polymer composites. Moreover, although brominated flame retardants have been banned or phased out in some countries and regions, while some new flame retardants have been developed as replacements, the brominated flame retardants still play a significant role in PCB development due to their extraordinary flame-resistant effects on combustible resins. Thus, traditional combustion of these non-metallic components in WPCB may induce the release of toxic gases, such as the dibenzodioxins, polybrominated dibenzofurans, and dioxins and furans [70,71,72]. In contrast, when the non-metallic components are further employed in polymer composites, the lifespan of a WPCB is effectively extended, and it does not pose a great threat to the environment due to it being present in low concentrations.

## 3. Reutilization of WPCB Non-Metallic Components in Sustainable Polymer Composites

Polymer composites are multiphase materials combined with polymer matrixes, fillers, and other additives, which can achieve superior properties to those of pure polymer matrixes. Based on the structure and characteristics of the non-metallic WPCB components mentioned above, the incorporation of WPCB non-metallic powders can replace some conventional fillers and enhances the comprehensive properties of polymer composites. Until now, the WPCB non-metallic components have been widely used in various polymer matrixes, such as thermosetting resins (e.g., epoxy resins, unsaturated polyester resin, phenolic resin, etc.), thermoplastics (e.g., polypropylene, poly(vinyl chloride), polyethylene, polycarbonate, acrylonitrile–butadiene–styrene, etc.), and rubber composites, which has sparked extensive attention [73]. In this section, the reutilization of WPCB non-metallic components in different polymer matrixes is comprehensively reviewed, and the structure–properties relationships between WPCB non-metallic components and polymer composites are critically analyzed.

### 3.1. Reutilization of WPCB Non-Metallic Components in Thermosetting Composites

**Figure 2:**
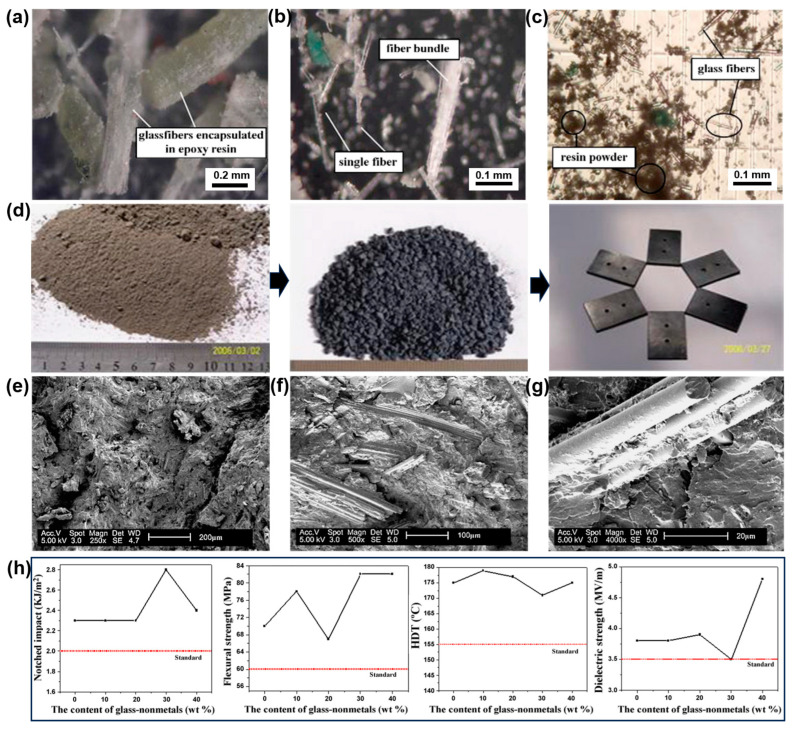
Micrographs of WPCB non-metals with different particle sizes: (**a**) 0.3–0.125 mm, (**b**) 0.125–0.07 mm, (**c**) <0.07 mm; (**d**) schematic illustration of the preparation of PMC filled with WPCB non-metals; SEM images of the fractured morphologies of (**e**) ordinary PMCs and (**f**,**g**) PMCs filled with WPCB non-metals; (**h**) properties of PMC with WPCB non-metals. Reprinted with permission from [75]. Copyright: 2008, Elsevier.

As one of the most common and oldest thermosetting resins, phenolic resin was usually used as a bonding agent to prepare phenolic molding compounds (PMC) via combination with various fillers, curing agents, and other functional additives under high temperatures and certain pressures [17]. And benefiting from the advantages of low cost, a simple manufacturing process, excellent mechanical properties, and high thermal stability, PMC was widely used in many areas, such as radios, kitchen appliances, and electronic switches [74]. Until now, the most-commonly used filler in PMC was wood powder, but with the gradual exhaustion of wood resources and the rising cost of wood powder, it is imperative to find alternative materials to reduce the cost of raw materials and effectively protect environmental resources. Recently, Guo et al. systematically studied the feasibility of recycling WPCB non-metallic components to create a replacement for wood flour in the manufacturing of PMC [74,75,76]. As illustrated in Figure 2a, it was observed that the obtained non-metals with particle sizes between 0.3 and 0.125 mm mainly consisted of fiber–particulate bundles, and most fibers are encapsulated using thermosetting resin. Additionally, both fiber bundles and single fibers can be observed in the non-metals, ranging from 0.125 to 0.07 mm (Figure 2b). With the particle further reducing to a size shorter than 0.07 mm, there were almost single glass fibers and thermosetting powders, as depicted in Figure 2c, which accounts for the highest proportion of 34.6%. The preparation diagram that describes PMC filling with WPCB non-metallic components was illustrated in Figure 2d. After the pre-mixing of phenolic resin, fillers, and other additives, the obtained mixture was compression-molded into testing samples under a high temperature and pressure. Generally, strong interfacial bonding between polymer matrix and fillers was closely related to the superior mechanical properties of PMCs. As depicted in Figure 2e, the flexural fractured morphology of a PMC filled with wood flour is smooth and exhibits deep gaps, while the glass fibers were bonded onto or embedded into the phenolic resin for the PMC filled with WPCB non-metals. The fiber pull-out phenomenon and interfacial debonding between phenolic resin and fillers were also observed (Figure 2f,g). A strong interfacial interaction can have a positive influence on the mechanical properties of PMC. As a result, when 40 wt% of non-metals were filled with phenolic resin, the flexural strength, impact strength, heat deflection temperature, and dielectric strength of as-prepared PMC reached 82 MPa, 2.4 kJ/m^2^, 175 °C, and 4.8 MV/m, respectively, which are superior to the national standard for PMC. Moreover, the curing behaviors of PMCs filled with WPCB non-metals were also investigated [77]. It was found that the incorporation of WPCB non-metals effectively reduces the curing activation energy of PMC based on the Kissinger model, which should be attributed to the impurities acting as a catalyzer. Consequently, it is a promising strategy for the achievement of the environmental protection and high value-added reutilization of WPCB non-metallic components by developing low-cost PMCs with excellent properties.

Epoxy resins (ERs) are another typical thermosetting polymer, and they have been extensively utilized in various applications, such as adhesives, coatings, electronic industries, automobiles, and fiber-reinforced composites, due to their outstanding mechanical, electrical, and chemical properties [78,79,80]. Yokoyama et al. explored the reuse of WPCB non-metals as the filler of epoxy resin, mainly investigating their mechanical properties [81]. It was found that their mechanical properties were adequate to serve as a construction material, but slightly lower than those of the reference samples containing silicon powder. It is worth noting that the surface modification of non-metals using an amino–silane coupling agent can effectively enhance their flexural strength. Mou et al. investigated the feasibility of using WPCB non-metals to manufacture composite boards, using the ERs as an adhesive [82]. The results indicate that the recovered WPCB non-metals can serve as a substitute for silica and talc. When 15 wt% of non-metallic powders were incorporated into ERs, their mechanical strength and modulus are effectively enhanced. Moreover, Chai et al. found that the incorporation of WPCB non-metals can not only enhance the flexural strength of EP composites, but also decrease their thermal expansion coefficient, whereas the insulation properties did not show obvious deterioration [83]. Thus, they can be further applied to produce the substrate used in a circuit board.

**Figure 3:**
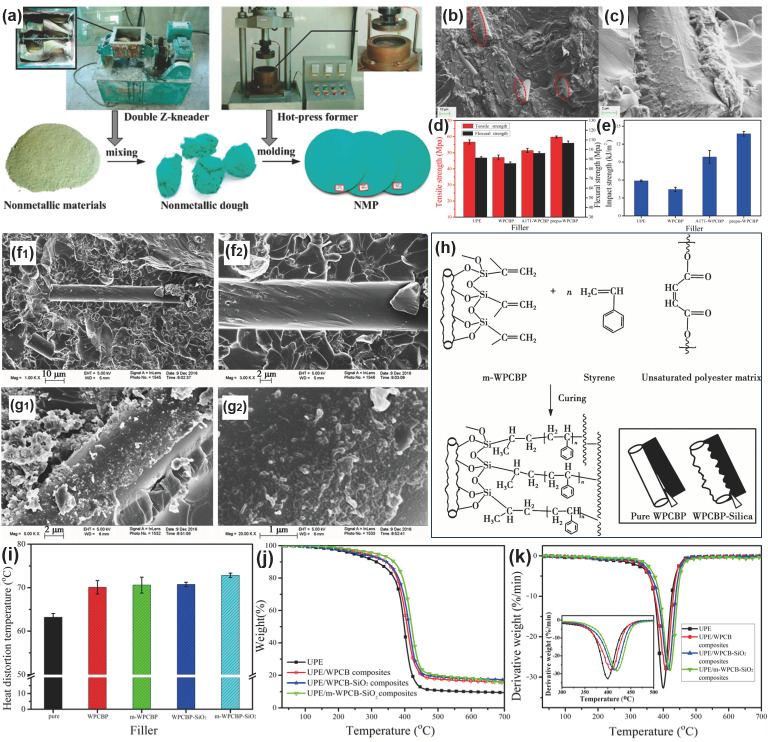
(**a**) Schematic illustration of the production of the NMP. Reprinted with permission from [88]. Copyright: 2008, American Chemical Society. (**b**,**c**) SEM images of the fractures of UPE/pre-PU–WPCB non-metal composites; (**d**) tensile strength and flexural strength of the UPE composites; (**e**) impact strength of the UPE composites. Reprinted with permission from [89]. Copyright: 2017, Wiley. SEM images of UPE composites filled with (**f1**,**f2**) WPCBP and (**g1**,**g2**) m-WPCBP-SiO_2_ hybrid filler; (**h**) proposed model of the reinforcing mechanism of UPE composites; (**i**) heat distortion temperature, (**j**) TGA, and (**k**) DTG curves of UPE and different UPE composites. Reprinted with permission from [90].

Unsaturated polyester resin (UPR) was also employed as a bonding agent because of its excellent processability, prominent chemical resistance, and low cost. And fiber-reinforced UPR composites have been widely utilized in various areas, such as the automobile, marine, and aerospace industries [84,85,86]. In order to make full use of the WPCB non-metals in UPR, Hong et al. first incorporated WPCB non-metal powder as a filler into unsaturated polyester resin and systematically explored the influence of the particle size and surface modification of non-metal powder on their mechanical and thermal properties [87]. It was found that adding WPCB non-metals can affect the free radical activity of unsaturated polyesters and reduce their curing rate. Additionally, the WPCB non-metals can endow the UPE with higher glass transition temperature and impact toughness. Subsequently, Guo et al. prepared a novel non-metallic plate (NMP) by employing unsaturated polyester resin as a bonding agent via the hot press process and explored the influence of the morphology and particle size of WPCB non-metallic powder on the performance of NMP [88]. The preparation process of the NMP was illustrated in Figure 3a. The results show that when the added amount of non-metallic powder is 20 wt%, and the NMP filled with non-metallic powder with a particle size of less than 0.07 mm exhibits outstanding mechanical properties, in which the bending strength reaches 68.8 Mpa, while the impact strength reaches 6.4 kJ/m^2^, thus meeting the performance requirements of park benches, fences, and other products. This method provides a possible means of achieving the sustainable reutilization of WPCB non-metallic materials and has promising prospects in terms of reducing the environmental pollution that occurs during PCB recycling. In our previous work, we firstly prepared a type of room temperature-cured UPE composite, using WPCB non-metals as the reinforcing filler [23]. The effects of the WPCB non-metal content on the mechanical properties and thermal stability of UPE composites were investigated. It was found that the incorporation of WPCB non-metallic components into UPE composites with appropriate content displayed more enhanced mechanical properties and heat distortion temperatures than the polymer matrix. Furthermore, the WPCB non-metals can significantly improve the thermal stability of UPE composites based on the analysis of thermal degradation kinetics. Furthermore, to strengthen the interfacial bonding between WPCB non-metals and UPE, we synthesized a polyurethane pre-polymer that was chemically bonded to the WPCB non-metals’ surfaces [89]. As shown in Figure 3b,c, the WPCB non-metallic components were widely wrapped via the UPE matrix and formed strong interfacial interactions with the UPE matrix, which exhibited a positive influence on the stress transfer and enhanced the mechanical performances of the UPE composites. Accordingly, the mechanical performances of the UPE/pre-PU–WPCB non-metal composites exhibited more remarkable enhancements than those of the UPE/WPCB non-metal composites; in particular, the impact toughness was enhanced three-fold (Figure 3d,e).

In fact, in addition to the surface modification using organic modifiers, the rational construction of novel hybrid fillers is also helpful to achieve remarkable properties of UPE composites. Typically, silica nanoparticles were chemically immobilized onto the WPCB non-metals surface via the sol–gel method to develop a novel WPCB–SiO_2_ hybrid filler [90]. As depicted in Figure 3f1,f2, the interfacial bonding between glass fiber and unsaturated polyester matrix is poor, and the glass fiber surface is smooth and basically exposed on the surface of the UPE resin matrix. While the UPE composites filled with modified WPCBP–SiO_2_ hybrid filler exhibit strong interfacial bonding (Figure 3g1,g2). It can be observed that a layer of resin was adhered to the surface of the hybrid filler, indicating that more molecular chains of unsaturated polyester could be restricted. Thus, when the composite was subjected to external force, the external stress could be effectively transferred to the hybrid filler, and the mechanical performances of the composites greatly improved (Figure 3h). Moreover, the construction of the WPCBP–SiO_2_ hybrid filler could effectively enhance the heat distortion temperature and thermal stability of the UPE (Figure 3i–k). The idea of constructing the hybrid filler prompted us to further explore the application of WPCB non-metallic components in flame-resistant materials. It was found that the combination of WPCB non-metals and halloysite nanotubes could endow the UPE composites with the dense carbon layer, excellent flame resistance, and thermal stability [91]. Moreover, the potential applications of the UPE composites filled with WPCB non-metals were explored. Typically, Cai et al. investigated the sound insulation properties of UPE/WPCB non-metal composites [92]. The results suggested that the maximum sound reduction index of WPCB-UP composites with a particle size < 0.71 mm reached 28.4 dB, showing great sound insulation application potential. Luo et al. recycled the WPCB non-metals to prepare bulk molding composites (BMCs), which were further utilized to manufacture composite manhole covers (CMCs) [93]. Compared to the traditional filler of CaCO_3_, the WPCB non-metals exhibited a higher compatibility with UPE. In particular, after performing surface modification using coupling agents, the BMC material showed strong interfacial bonding and enhanced mechanical properties. The constant loading test, together with ANSYS finite element analysis, was conducted to confirm the application feasibility of the prepared CMC. Based on the above discussion, it can be found that the WPCB non-metals can not only serve as a reinforcing filler to enhance the mechanical performances of thermosetting resins, but also play a significant role in the functionalization of thermosetting resins, which have favorable economic and social benefits and offer a guarantee of the practical industrial application of WPCB non-metallic components.

### 3.2. Reutilization of WPCB Non-Metallic Components in Thermoplastic Composites

**Figure 4:**
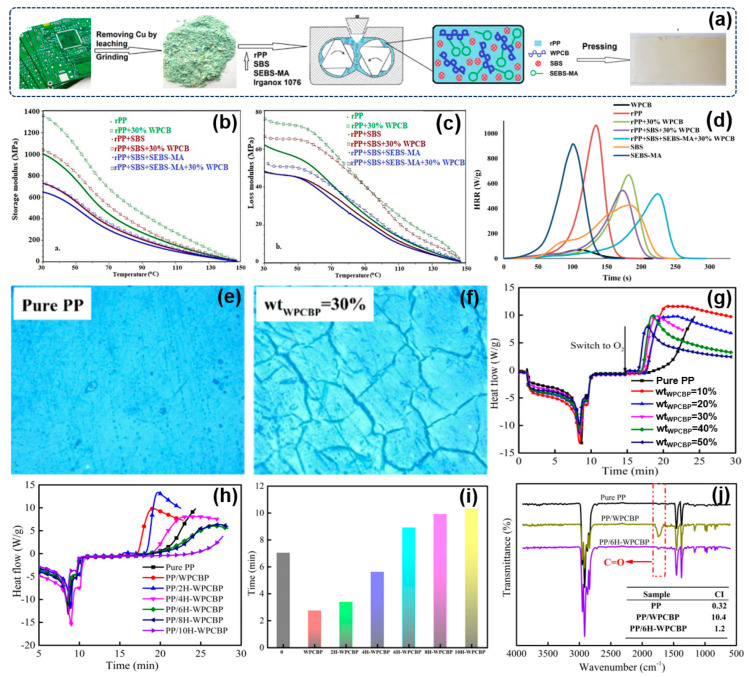
(**a**) Schematic presentation of the process of PP composites; (**b**) storage modulus and (**c**) loss modulus of rPP composites; (**d**) HRR dependence on the time recorded for rPP, WPCB, and their elastomeric composites. Reprinted with permission from [106]. Copyright: 2020, Elsevier. Digital images of (**e**) pure PP and (**f**) PP/WPCB non-metals composites after thermal oxidative aging; (**g**) OIT curves of PP composites containing different amounts of WPCBP; (**h**) OIT curves and (**i**) values of different PP composites; (**j**) FTIR spectra of PP and PP composites after aging. Reprinted with permission from [107]. Copyright: 2019, Wiley.

Polyolefin is a general term for a class of thermoplastic resin obtained via the individual or copolymerization of small molecules (ethylene, propylene, 1-butyene, 1-pentene, 1-hexene, 1-methyl-1-pentene, α-oleene, and some cyclic olefins), which have been widely used in agriculture, packaging, electronics, automotive, machinery, and daily necessities due to their moderate cost, easy processing and molding, excellent comprehensive performance, etc. Typically, polypropylene is one of the most important commodity general thermoplastics, having excellent mechanical properties and heat resistance [94,95,96,97]. However, in comparation to the engineering plastics, the PP still suffer from lower strength and high notch sensitivity. To expand the applications of PP, various inorganic rigid fillers have been introduced into PP matrixes to effectively improve their properties or reduce their cost, which has provoked wide interest in the past few decades [98,99,100]. Recently, Zheng et al. have systematically investigated the feasibility of reutilizing the WPCB non-metals to reinforce the PP composites [101,102,103,104]. It was found that both tensile and flexural performances of PP composites were greatly enhanced by incorporating the WPCB non-metallic components. The maximum improvement in the tensile strength, flexural strength, and flexural modulus of the PP composites reached 28.4%, 87.8%, and 133.0%, respectively [35]. The results of the vicat softening temperature indicates that the WPCB non-metals improve the thermal resistance of PP composites. Moreover, the particle size and amount can affect the final properties of PP composites. To further strengthen the interfacial compatibility between WPCB non-metallic particles and PP matrix, the WPCB non-metallic particles were chemically modified using a KH–550–silane coupling agent, which prevented and delayed the extension of cracks and resulted in the enhanced strength and rigidity of PP composites. Moreover, the WPCB non-metals were modified using calcium pimelate (PA) and blended with PP via a melt-blending strategy [105]. It was suggested that when cooling and crystallizing from the melt, β-PP was obtained due to the surface effects of PA. Compared to the neat PP, the impact property and flexural modulus of PP composites filled with 10 wt% of PA modified non-metals were increased by 205.3 and 61.8%, respectively. In addition to the reinforcing effect, the WPCB non-metals can serve as a fire resistance modifier. As shown in Figure 4a, Grigorescu et al. blended the WPCB non-metals with recycled polypropylene and styrene–butadiene block copolymers, investigating their dynamic–mechanical properties and flammability [106]. The functionalized block-copolymer containing maleic groups can greatly increase the interfacial compatibility of WPCB non-metals with polypropylene. Figure 4b shows the temperature dependence of the storage (E′) of rPP composites. An increase of 35% was achieved for rPP/30% WPCB composites relative to the rate of the control samples, whereas a 42% increment was obtained for the rPP/WPCB composites containing SBS. The loss modulus (E″) dependence on temperature is depicted in Figure 4c. By introducing the elastomers, the E″ of samples decreases, while the E″ increases with the rigid filler. A shift in the maximum peak can be ascribed to the immobilization of the polymer chains at the interphase with WPCB non-metals. In particular, the samples containing elastomers and 30% WPCB non-metals show the highest flame resistance, having a heat release capacity of 522 J/(g·K) and char yield of 18.75%. In comparison to rPP, an apparent decrease of 51.19% in heat release capacity can be achieved for rPP/SBS/SEBS–MA/WPCB composites due to the strong interfacial contact. As expected, the WPCB non-metals can improve the fire resistance of rPP to some extent.

Although the above results indicate that the reutilization of WPCB non-metals in PP composites is a promising means of achieving resource recycling and low environmental pollution, some challenges still affect these sustainable PP composites. Typically, the residual multivalent transition metals (e.g., Cu and Fe) in WPCB powders accelerate the degradation of PP molecule chains, thus limiting their wide practical applications. As illustrated in Figure 4e,f, the digital images of PP/WPCB non-metals composites after thermal oxidative aging showed more cracks of different sizes than pure PP, demonstrating that WPCB non-metals had a terrible thermal oxidative aging effect on PP composites [107]. Generally, the oxidation induction time (OIT) could effectively reflect the oxidative properties of polymer composites; the higher the exhibited resistance to thermal oxidation, the higher the OIT value. It is clearly seen in Figure 4g that the OIT values dramatically decreased after the incorporation of WPCBP owing to the catalytic degradation effect of multivalent transition metals. To enhance the weathering properties of PP composites, Tian et al. further treated the WPCB non-metals using nitric acid. Figure 4h shows the OIT curves and values of PP composites containing nitric acid-treated WPCBPs. In comparison to the PP/WPCBP composites, the nitric acid-treated WPCBPs could endow the PP composites with higher OIT values. In particular, when the concentration was higher than 4 wt%, the OIT values of PP composites were higher than those of pure PP, indicating that the WPCBP without multivalent transition metals may serve as an antioxidant to enhance the anti-aging performance of the PP composite. Moreover, the FTIR results of PP and PP composites further demonstrated the OIT analysis. The carbonyl peak of PP/6H–WPCBP composites that originated in the thermal oxidation process show decreases more significant than that those of PP/WPCBP composites.

**Figure 5:**
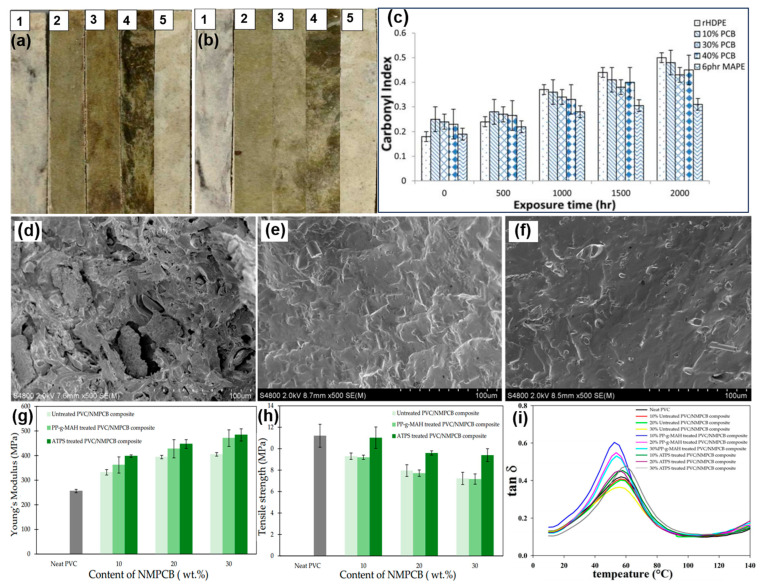
Digital images of flexural samples (**a**) before weathering and (**b**) after 2000 h of accelerated weathering; (**c**) carbonyl index of unfilled rHDPE and rHDPE/PCB composites after accelerated weathering. Reprinted with permission from [109]. Copyright: 2015, Wiley. SEM images of PVC composites with (**d**) untreated 30 wt% NMPCB; (**e**) 30 wt% NMPCB-treated ATPS; (**f**) 30 wt% NMPCB-treated PP-g-MA; (**g**) Young’s modulus of PVC/NMPCB composites; (**h**) tensile strength of PVC/NMPCB composites; (**i**) influence of interfacial modifiers on the loss factor or tan δ of PVC/NMPCB composites. Reprinted with permission from [116]. Copyright: 2023, MDPI.

Polyethylene is another important thermoplastic with excellent mechanical properties and chemical resistance. Muniyandi et al. produced a sustainable rHDPE/PCB composite by combining the WPCB non-metallic powder and recycled HDPE (rHDPE), and the authors evaluated their mechanical, thermal, and leaching properties [108]. The results showed that the incorporation of a 6-parts-per-hundred-resin MAPE improved the flexural strength, tensile strength, and impact strength by 71%, 98%, and 44%, respectively, relative to those of the unmodified composites. Moreover, the concentrations of Cu and Br in the leachates from the rHDPE/PCB composites were far below the regulatory level. Although the rHDPE/PCB composite had excellent physical properties, the weathering properties of composites were rarely considered. Thus, Muniyandi et al. investigated the changes in the structure and properties of the rHDPE/PCB composites under accelerated weathering exposure to evaluate the suitability of composites for outdoor applications [109]. As illustrated in Figure 5a,b, the appearances of the rHDPE and rHDPE/PCB composites after 2000 h of exposure indicated that all of the composites became lighter after accelerated weathering exposure, and their surfaces became rough and powdery. Figure 5c depicts the carbonyl index (CI) of the rHDPE and rHDPE/PCB composites during the accelerated weathering process. The results suggested that the CI improves with a longer exposure time, indicating that the oxidation reaction continuously proceeded during the aging process. Moreover, rHDPE/PCB composites compatibilized with MAPE had a relatively lower CI than that of the composites without compatibilizer. Yang et al. also studied the thermal oxidative aging performances of HDPE/WPCB non-metals composites and demonstrated that the WPCB non-metals has a similar oxidation resistance to the two other commercial fillers [110]. Similarly, the WPCB non-metals have been utilized in polyvinyl chloride (PVC) [111,112,113]. Grigorescu et al. demonstrated that a 15–20% non-metallic fraction of WPCB can endow the recycled PVC with properties suitable for reuse as an insulating technical material [114]. Wang et al. found that the diameter and contents of glass fiber recovered from WPCB determined the tensile and bending strengths of as-prepared PVC composites [115]. It can be expected that the interfacial compatibility between WPCB non-metals and polymer matrixes was significant for the final comprehensive properties of polymer composites. Consequently, Moe et al. explored the influence of interfacial modifiers, namely polypropylene-grafted-maleic anhydride (PP-g-MAH) and γ-aminopropyltriethoxysilane (ATPS), on the properties of PVC/WPCB composites [116]. As depicted in Figure 5e,f, the PVC/WPCB composites without interfacial agents showed obvious voids around the WPCB and holes caused by glass fiber pullout due to weaker interfacial bonding between the PVC matrix and fillers. The surface modification of WPCB non-metals using ATPS and PP-g-MAH can improve the NMPCB dispersion and interfacial adhesion, as well as reduce the formation of voids and holes. As a result, the as-obtained PVC/NMPCB composites containing modifiers exhibited excellent tensile strengths and moduli in comparison to the unmodified PVC/NMPCB composites (Figure 5g,h). Furthermore, the shifting of the loss factor for PVC/NMPCB composites with different interfacial agents illustrates the enhanced interfacial bonding between the NMPCB and PVC matrixes (Figure 5i).

**Figure 6:**
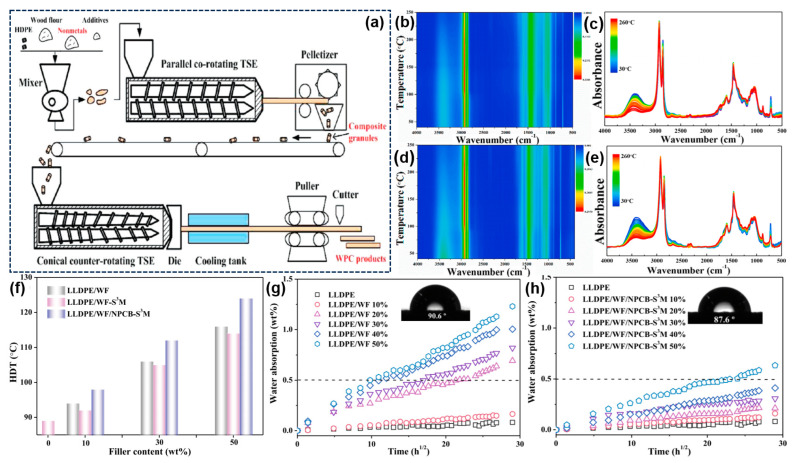
(**a**) Flowchart detailing the preparation of WPC products. Reprinted with permission from [25]. Copyright: 2010, American Chemical Society. In situ FTIR contour map and the corresponding spectra for the (**b**,**c**) LLDPE/WF composites and (**d**,**e**) LLDPE/WF/NPCB−S_3_M composites; water absorption by the (**f**) LLDPE/WF and (**g**) LLDPE/WF/NPCB−S_3_M composites; (**h**) heat deflection temperature of the LDPE composites. Reprinted with permission from [124]. Copyright: 2020, Elsevier.

Wood–plastic composites (WPC) are generally produced by blending thermoplastics, wood flour, and small amounts of additives, and they have been extensively used in outdoor decoration, garden architecture, industrial flooring, landscape timbers, automobile paneling, and furniture due to their non-formaldehyde, light weight, abundance, and other characteristics [117,118,119]. Compared to the wooden products, the WPC is durable and requires low maintenance; therefore, it is ideal for use in unstructured applications. Among these applications, the wood flour is one of the most common fillers used in WPC, as it reduces costs and improves the mechanical strength of WPC. With the increased demand for WPC, developing other non-wood resources is essential to meeting the raw material requirements and protecting timber resources [120,121,122,123]. Inspired by this fact, Guo et al. innovatively prepared a new kind of WPC by compounding WPCB non-metals, recycled HDPE, wood flour, and other additives, as shown in Figure 6a [25]. It was found that adding WPCB non-metals to WPC effectively enhanced the flexural and tensile properties and synergistically decreased the screw withdrawal strength. When 40 wt% of non-metals were added, the flexural strength, tensile strength, and impact strength reached 23.4 MPa, 9.6 MPa, and 3.03 J/m^2^, respectively. Moreover, Yang et al. developed a novel strategy to prepare WPC-reinforced with NPCB via the solid-state shear milling (S_3_M) process [124]. The results revealed that S_3_M treatment can effectively exfoliate NPCB into high-aspect ratio glass fibers. And the as-prepared WPC based on S_3_M process showed uniform filler dispersion and strong interfacial bonding between the fillers and linear low-density polyethylene (LLDPE). Accordingly, the tensile strength of WPC reached 32.4 MPa, while the storage modulus showed a high value of 616 MPa, even at 100 °C, thus efficiently extending the maximum service temperature of the WPC. In addition, to explore the thermal behavior of the WPC, the in situ FTIR curves of the LLDPE/WF composites and LLDPE/WF/NPCB-S_3_M composites were performed, and they are displayed in Figure 6b–e. The results showed no characteristic absorption peak of the carbonyl group appeared during the heating process, illustrating that the WPC molecular chain is very stable during melt processing. The outstanding thermal stability of the LLDPE/WF/NPCB-S_3_M composites may be ascribed to the biobased phenols derived from the wood flour, which may act as antioxidants in the WPC. The HDT results indicated that the NPCB-reinforced WPC exhibited a higher HDT (112 °C) than the pure LLDPE (89 °C) (Figure 6f) owing to the strong skeleton structure of glass fiber in NPCB. Moreover, as mentioned above, the WPCs are usually utilized in outdoor applications, which require superior water resistance to meet the service lifespan of the WPC. As displayed in Figure 6g, the WPC with 30-percentage-by-weight wood content showed a higher water absorption (0.77%) than pristine LLDPE because the interfacial gaps between the wood and polymer can result in the penetration of water molecules into LLDPE/WF composites. In contrast, the water absorption of the NPCB-reinforced WPC was significantly reduced to 0.21% (Figure 6h), exceeding the values previously reported for the NPCB-reinforced WPC, which should be attributed to the good filler dispersion and the fewer voids present within the composites. Additionally, the presence of fewer hydroxyl groups in the NPCB than in the wood flour is beneficial, as it leads to lower water absorption. Notably, the organic pollutants and heavy-metal concentrations are basically negligible, which are far below the regulatory limits used to identify hazardous waste. Thus, the reuse of NPCB in high-strength WPC is a feasible choice in terms of both relieving environmental pollution and producing high value-added products.

**Figure 7:**
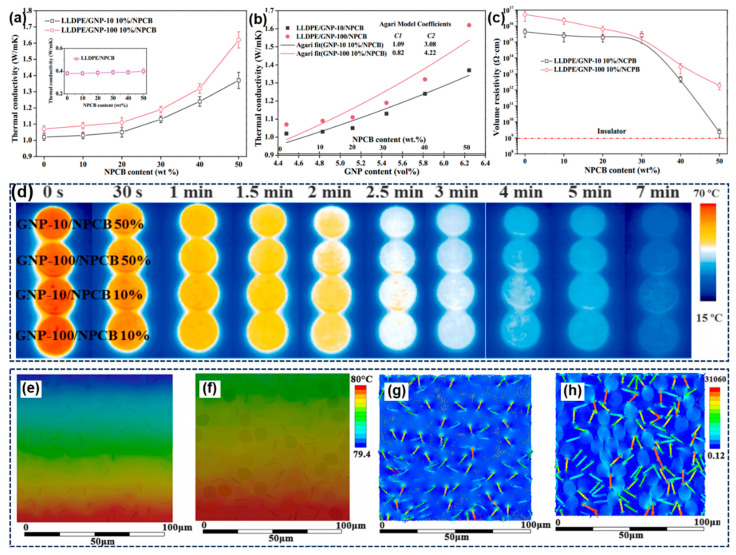
(**a**) Thermal conductivity and (**b**) the Agari model fitting curves of LLDPE composites; (**c**) volume resistivity of composite with various NPCB contents; (**d**) infrared thermal images of different samples; temperature and heat flux distributions of LLDPE/GNP (**e**,**g**) and LLDPE/GNP/NPCB (**f**,**h**) composites in finite element models. Reprinted with permission from [125]. Copyright: 2020, Elsevier.

It is worth noting that the WPCB non-metals also play a crucial role in the functionalization of thermoplastic composites. Yang et al. innovatively explored the possibility of employing NPCB to prepare thermal management materials [125]. Benefiting from the high aspect ratio of NPCB, the tensile strength of LDPE/graphite nanosheets (GNP)/NPCB composites improved from 24.9 MPa to 36.2 MPa. As shown in Figure 7a, the thermal conductivity of LDPE/GNP/NPCB composites gradually increased with the increase in the NPCB content by forming more effective thermally conductive pathways. In particular, when incorporating 50 wt% of the NPCB, the thermal conductivity of GNP-10- and GNP-100-filled composites were enhanced by 34% and 52%, respectively, reaching 1.4 W/m·K and 1.6 W/m·K, respectively, thus demonstrating the synergistic effect between GNP and NPCB, which improves the thermal conductivity of the composites. Moreover, it can be noticed that the C1 values of composites are close to each other, whereas the fitting coefficients of C2 greatly deviate from the Agari model and are much larger than 1, indicating that a more efficient thermal conduction network is formed in the GNP-100-filled composites. Furthermore, the electrical conductivity of all samples is higher than 109 Ω·cm, meeting the requirements of insulating and thermally conductive materials. To evaluate the cooling process of as-prepared composites in thermal management applications, the surface temperature distribution was recorded via an infrared thermal camera. It was found that the NPCB-reinforced composites exhibited lower surface temperatures and showed superior thermal dissipation capabilities. Furthermore, finite element simulation was performed to visually reveal the heat flow diffusion and offer a helpful understanding of the thermal conduction process of composites. The results illustrate that the thermal propagation in the filler network of composites is in close agreement with the classical Agari model. The GNP network is mainly responsible for the efficient thermal diffusion and the increased thermal conductivity of composites. In addition to above-mentioned general thermoplastics, the WPCB non-metals have been widely applied in some recycled engineering thermoplastics, such as acrylonitrile–butadiene–styrene (ABS) and polycarbonate (PC) [126,127]. For example, Sun et al. developed a sustainable ABS composite by combining two waste resources derived from recycled ABS waste plastic and WPCB non-metals. It was revealed that the WPCB non-metals significantly enhanced the mechanical performances of ABS composites. Typically, 30 wt% of non-metal particles can obtain a flexural strength of 72.6 MPa, a flexural modulus of 3.57 GPa, and an impact strength of 15.5 kJ/m^2^ [126]. This method represents a promising strategy to use in dual e-waste recycling and relieving the environmental pollution. Notably, despite various feasible applications of WPCBs’ non-metallic components in thermoplastics, there are still some challenges. For example, many thermoplastics have a poor weathering properties and are sensitive to heat, UV exposure, and oxidation, which restricts their industrial applications. Consequently, enhancing the recovery efficiency of residual metal and preparing effective antioxidants should be given greater attention in future studies.

### 3.3. Reutilization of WPCB Non-Metallic Components in Rubber Composites

**Figure 8:**
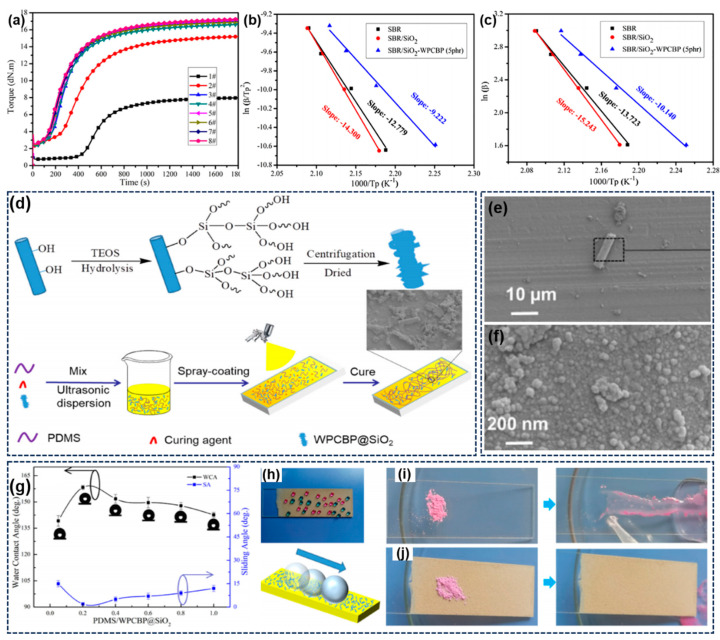
(**a**) Vulcanization curves of SBR compounds; linear fitting used to calculate E_a_ via the (**b**) Ozawa and (**c**) Kissinger method. Reprinted with permission from [73]. Copyright: 2020, Wiley; (**d**) schematic presentation of the fabrication of WPCBP@SiO_2_ and PDMS/WPCBP@SiO_2_ coatings; (**e**,**f**) SEM images of WPCBP@SiO_2_ hybrid fillers with different magnification values; (**g**) water contact and sliding angles of PDMS/WPCBP@SiO_2_ coatings; (**h**) digital images of water droplets on the PDMS/WPCBP@SiO_2_ coating; self−cleaning photographs of (**i**) blank glass and (**j**) PDMS/WPCBP@SiO_2_ coatings. Reprinted with permission from [134]. Copyright: 2021, Wiley.

Until now, the main industrial applications of WPCB non-metals have been thermosetting resins and thermoplastics, as mentioned above. It is also be noting that rubbers, as strategically important polymer materials, need to be reinforced with various fillers to achieve wide practical applications owing to their poor mechanical properties [128,129,130]. In particular, in the past few years, driven by environmental pressures, some industrial waste and by-products, including fly ash, marble slag, and agricultural waste, have been used as alternatives to commercial fillers in rubber composites, which give great hope for the high value-added applications of WPCB non-metals [131,132,133]. Therefore, in our previous work, the WPCBP was first introduced into styrene–butadiene rubber (SBR) composites to systematically investigate its potential as a curing additive and reinforcing filler of rubber composites [73]. As shown in Figure 8, the vulcanization behaviors and kinetics of SBR compounds were studied using a moving-die rheometer and differential scanning calorimeter. It was found that the curing rate was suppressed after the incorporation of SiO_2_, while the addition of a small content of WPCBP (5 phr) could evidently accelerate the crosslinking process. In general, the acidic hydroxyl groups on the surface of SiO_2_ could adsorb some basic accelerators, and the diffusion and accessibility of curing agents to the rubber vulcanization sites were also limited due to the severe aggregate of SiO_2_ via hydrogen–bond interactions. However, the incorporation of WPCBP could endow the rubber composites with a higher curing rate. This outcome may be ascribed to the activation effect of metal oxide and the accelerating effect of residual additives in WPCBP. Furthermore, to more accurately explore the nature of vulcanization process, the vulcanization kinetics of SBR compounds was investigated using the Kissinger and Ozawa method. The results suggested that the SBR composites filled with WPCBP exhibited lower values of exothermal peak temperature (T_p_) and activation energy (E_a_), dramatically improving the curing rate of SBR compounds, which is consistent with the results of the curing curves. As discussed above, WPCBP mainly consisted of resin powders and glass fibers, and they contained a little residual metal oxide and additives. Generally, the inorganic metal derivatives (eg, ZnO, MgO) are suitable for use as curing activators in rubber compounds to improve their curing efficiency. Moreover, some residual additives may have the same active groups as the commercial rubber accelerator, which results in the lower activation energy of SBR/SiO_2_/WPCBP. Subsequently, Liu et al. reported a superhydrophobic functional coating based on WPCBP and PDMS silicone rubber inspired by the lotus leaf effect [134]. As shown in Figure 8d, the surface of WPCBP first formed a layer of silica nanoparticles. The received WPCBP@SiO_2_ hybrid filler was combined with PDMS to develop a novel low-cost and environmentally friendly superhydrophobic coating, providing a significant platform for the sustainable high-valued reuse of WPCBP in superhydrophobic composites. Unlike the WPCBP, it can be obviously observed that the prepared WPCBP/SiO_2_ hybrid filler had a rough surface structure, as depicted in Figure 8e,f, the higher roughness of which is beneficial to the construction of micro-nano structures used in superhydrophobic coatings. As presented in Figure 8g, when the ratio of PDMS and WPCBP@SiO_2_ is 0.2, the WCA of as-prepared coatings exhibits the highest value of 158°, and the SA is only 2.0°, thus showing superior superhydrophobic properties. Moreover, unlike the initial glass slide, the as-prepared PDMS/WPCBP/SiO_2_ coatings show excellent self-cleaning performance, with the pink chalk powders being automatically removed using the rolling water droplets, leaving a clean and dry surface. In particular, these low-cost superhydrophobic coatings can be applied to wood buildings or municipal trash cans, which can efficiently prevent the substrate from experiencing deformation and damage in humid environments, thus showing important practical value in terms of reducing environmental pollution and developing low-cost superhydrophobic coatings.

## 4. Summary, Challenges, and Outlook

In this review, we summarized and discussed recent advances in sustainable polymer composites based on WPCB non-metallic components, and emphasis was placed on the preparation, structure, and properties of these polymer composites, as well as their potential applications. It has been demonstrated that the WPCB non-metals can serve as a promising reinforcing and functional filler to construct various ecofriendly polymer composites owing to their wide availability, low cost, high mechanical performances, and environmental nature. The incorporation of WPCB non-metallic powder could significantly ameliorate some physical and chemical properties of polymer composites, such as excellent mechanical performances, enhanced thermal stability, and flame resistance. In summary, significant advances have been achieved in the past few years, which have opened a new and feasible pathway for the high value-added reutilization of WPCB non-metallic components. However, there are still some scientific and technical issues that need to be resolved prior to their widespread adoption for practical applications. Efforts should be devoted to the following issues:

(1) The WPCB non-metals are diverse and complex in terms of type, size, shape, components, and composition, typically including cured thermosetting resins, glass fiber (cellulose paper), ceramics, BFRs, residual metals, and other additives. Thus, the recovery process of WPCBs non-metallic components is also very complicated. In general, the methods of recovering non-metallic components from WPCB include physical recycling methods and chemical recycling methods. Physical recycling of the WPCB non-metals is a promising environmentally friendly recycling method that requires appropriate equipment investment and low energy costs. More work should be performed to explore comprehensive and industrialized application of the recovered WPCB non-metals through physical methods. However, there are still some issues related to the physical process of recycling WPCBs. It is generally very difficult to completely remove all metals through the physical recycling process, and some residual metals in the as-reclaimed non-metallic components may lead to the serious deterioration of the aging properties of polymer composites. Moreover, other than the glass fibers, residual metals, and resin powders, the influence of other metal derivatives and additives on the structures and properties of polymer composites is also worth studying in depth.
(2) Many studies have indicated that the addition of WPCB non-metallic components to polymer composites can indeed effectively reduce the cost of polymer composites and enhance their comprehensive properties to some extent, which is also critically significant in terms of relieving the environmental pollution caused by inappropriate traditional recycling methods. However, it is still challenging to achieve the strong interfacial interaction between polymer matrix and unmodified WPCB non-metals, causing the superiority of WPCB non-metals with a high aspect ratio not to have been fully exploited. Moreover, many thermoplastics and rubbers have poor weathering properties and are sensitive to heat, UV exposure, and oxidation, whereas some residual metals in the as-reclaimed non-metallic components can further aggravate the deterioration of the aging properties of polymer composites, which severely limits their widespread use, especially in outdoor applications. Consequently, it is highly advisable to develop and introduce some novel interfacial modifiers and antioxidants in polymer composites to further optimize the interfaces and endow the polymer composites with longer service lives. Moreover, the reliability and potential environmental risks of as-prepared sustainable composites need to be taken into account because of the residual metals and bromide flame retardants.
(3) Although the high value-added reutilization of WPCB non-metals in polymer composites has received a series of impressive advances, the recovery problem associated with WPCB non-metals has not been fundamentally solved, especially for cured thermosetting composites, which only extend the service life to a certain extent. In fact, if the thermosetting resin in PCB can be effectively degraded into small molecules or dissolved in some solvents, the recycling process of WPCB would become very simple, which is also in line with the strategy goals of “carbon peaking and carbon neutrality”. Therefore, it is not only necessary to carry out research into the high-value resource utilization of WPCB non-metals, but also to pay attention to the development of sustainable polymer matrixes, such as natural bio-based polymers, degradable synthetic polymers, etc. Moreover, this approach is expected to introduce the dynamic chemical bonds into thermosetting resins to prepare recyclable thermosets through molecular design. There is also an urgent need to develop a new generation of environmentally friendly bio-based additives, such as bio-based flame retardants, curing agents, antioxidants, etc. In conclusion, by systematically summarizing the relevant advances and investigating their preparation–structure–properties–applications relationships, we hope that this work can offer meaningful insights regarding the high value-added reutilization of WPCB non-metals in polymer composites.

## Data Availability

Not applicable.
